# Melatonin as a Redox Modulator in Developmental Programming: Implications for Cardiovascular–Kidney–Metabolic Risk

**DOI:** 10.3390/ijms27052390

**Published:** 2026-03-04

**Authors:** Chien-Ning Hsu, You-Lin Tain

**Affiliations:** 1 Department of Pharmacy, Kaohsiung Municipal Ta-Tung Hospital, Kaohsiung 801, Taiwan; cnhsu@cgmh.org.tw; 2 Department of Pharmacy, Kaohsiung Chang Gung Memorial Hospital, Kaohsiung 833, Taiwan; 3 School of Pharmacy, Kaohsiung Medical University, Kaohsiung 807, Taiwan; 4 Department of Pediatrics, Kaohsiung Chang Gung Memorial Hospital, Kaohsiung 833, Taiwan; 5 Doctoral Program of Clinical and Experimental Medicine, National Sun Yat-sen University, Kaohsiung 804, Taiwan; 6 College of Medicine, Chang Gung University, Taoyuan 333, Taiwan

**Keywords:** oxidative stress, melatonin, antioxidants, cardiovascular-kidney-metabolic syndrome, developmental origins of health and disease (DOHaD), nitric oxide

## Abstract

Melatonin, a multifunctional hormone with antioxidant, anti-inflammatory, and chronobiotic effects, is essential for a healthy pregnancy and fetal development. In the context of the Developmental Origins of Health and Disease (DOHaD), excessive oxidative stress acts as a key driver of maladaptive fetal programming, increasing lifelong susceptibility to cardiovascular, kidney, and metabolic (CKM) disorders. Importantly, most evidence derives from rodent models, and the protective effects of maternal melatonin supplementation appear partial and model-dependent rather than universal. Experimental studies indicate that maternal melatonin supplementation can prevent programmed hypertension, renal dysfunction, and metabolic derangements by restoring redox homeostasis, influencing epigenetic and nutrient-sensing pathways, and modulating the gut microbiome. Early clinical investigations in pregnancies complicated by preeclampsia or intrauterine growth restriction suggest that melatonin is well tolerated, improves placental function, and benefits neonatal outcomes. However, optimal dosing and long-term safety for offspring remain to be established. This review synthesizes mechanistic and translational evidence, framing melatonin as an integrative biological mediator with potential to guide preventive strategies and mitigate the intergenerational risk of CKM syndrome.

## 1. Introduction

In the current global health landscape, the escalating burden of chronic non-communicable diseases (NCDs) poses a substantial challenge to healthcare systems worldwide [1]. Several major NCDs converge within the framework of Cardiovascular–Kidney–Metabolic (CKM) syndrome, a systemic disorder formally defined by the American Heart Association in 2023 [2]. This construct underscores the tightly interwoven pathophysiological links among metabolic risk factors—including obesity and diabetes—chronic kidney disease (CKD), and cardiovascular disease (CVD) [3]. Emerging evidence suggests that up to 90% of adults in the United States may exhibit at least one stage of CKM syndrome [4], with a substantial proportion progressing to multiorgan dysfunction and increased mortality. Although conventional strategies have predominantly focused on interventions in adulthood, CKM syndrome may originate early in life, underscoring early life prevention—rather than late-stage management—as a critical unmet need [5].

Central to understanding these chronic conditions is the Developmental Origins of Health and Disease (DOHaD) framework, also referred to as life course theory [6,7,8]. This paradigm posits that a suboptimal intrauterine environment—shaped by maternal nutritional imbalance, systemic illness, or exposure to environmental toxins—induces structural and functional adaptations in the developing fetus that enhance short-term survival. Although initially advantageous, these adaptations predispose individuals to increased susceptibility to chronic diseases across the life span, a process known as developmental programming [9]. Importantly, the inherent plasticity of early development also provides a critical window for “reprogramming”, whereby early life interventions may reverse or mitigate these maladaptive trajectories before overt clinical disease manifests [10].

Oxidative stress is a central mechanism underlying developmental programming [11,12], resulting from an imbalance between reactive oxygen and nitrogen species (ROS/RNS) and endogenous antioxidant defenses. While physiological levels of ROS are essential for normal pregnancy processes, the fetus has limited antioxidant capacity and is therefore highly susceptible to excessive oxidative stress under adverse in utero conditions. Excess ROS can damage DNA, lipids, and proteins, leading to mitochondrial dysfunction and disrupted renal, metabolic, and cardiovascular development. In this context, antioxidant-based interventions have emerged as promising reprogramming strategies for the prevention of CKM syndrome [13].

Melatonin (N-acetyl-5-methoxytryptamine) is a potent antioxidant of growing interest [14,15]. Beyond circadian regulation, it is a pleiotropic hormone with antioxidant and anti-inflammatory properties, capable of crossing biological barriers and accumulating in mitochondria [16]. During gestation, maternal and placental melatonin help maintain placental redox homeostasis and provide circadian cues to the fetus [17]. Its antioxidant actions involve direct radical scavenging, activation of endogenous antioxidant enzymes, suppression of pro-oxidant pathways, and emerging epigenetic regulation during critical windows of organ development [18,19,20].

Animal studies provide strong proof-of-concept for melatonin as a reprogramming agent; maternal melatonin supplementation prevents programmed hypertension, CKD, and metabolic disorders across multiple experimental models and improves offspring outcomes [13]. However, translation to clinical practice remains limited. Although melatonin has a favorable safety profile and preliminary trials in pregnant women with preeclampsia suggest potential benefit, robust randomized controlled trials evaluating long-term offspring outcomes and optimal dosing during pregnancy are still lacking [21,22].

This narrative review synthesizes current evidence on the role of melatonin during pregnancy and its impact on the developmental programming of offspring health. Within the framework of CKM syndrome, we explore the molecular mechanisms—ranging from protection against oxidative stress to modulation of the gut microbiota—through which melatonin may exert beneficial effects. We further critically evaluate ongoing clinical trials and identify key knowledge gaps that must be addressed to translate melatonin from an experimental reprogramming agent into a clinically viable intervention. By integrating mechanistic and translational evidence, this review highlights the potential of melatonin to reduce the global burden of CKM syndrome and preserve long-term offspring health.

## 2. Oxidative Stress, Antioxidants, and the DOHaD Concept

### 2.1. The DOHaD Framework and Offspring Health

Research in the DOHaD field was pioneered by David Barker, who demonstrated that low birth weight is associated with an increased risk of coronary artery disease and hypertension in adulthood [23]. The Dutch Hunger Winter, a severe famine in the German-occupied Netherlands during the winter of 1944–1945, has become a seminal example in DOHaD research, offering a natural experiment to examine the long-term effects of maternal malnutrition during critical developmental windows. Offspring exposed to the famine in utero exhibited a higher prevalence of CKM-related phenotypes in adulthood, including obesity, type 2 diabetes, coronary artery disease, and hypertension [24].

**Figure 1:**
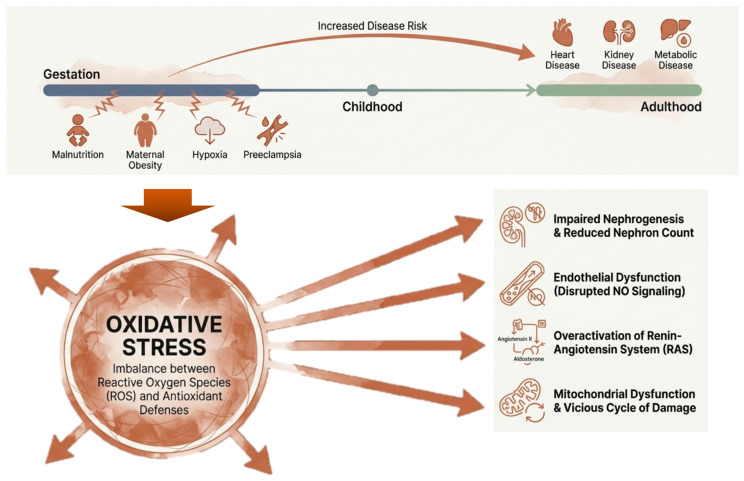
Interrelationships between early life insults, oxidative stress, and the subsequent development of cardiovascular–kidney–metabolic syndrome across the lifespan.

Evidence from mother–child cohorts further indicates that maternal undernutrition, obesity, smoking, and gestational hypertension are significant predictors of adult-onset CKM-related phenotypes in offspring [25,26,27]. Animal studies corroborate these findings, showing that early life insults such as overnutrition, metabolic disturbances, maternal illness, placental or hypoxic stress, chronodisruption, and drug or toxin exposure contribute to the developmental programming of CKM syndrome [28,29,30,31]. Notably, these diverse insults converge on common molecular pathways, particularly oxidative stress [32], which heightens offspring susceptibility to CKM syndrome across the lifespan (Figure 1).

### 2.2. Oxidative Stress as a Central Hub

Oxidative stress serves as a key mechanistic hub in the mechanisms driving developmental programming. It arises when the generation of ROS/RNS exceeds the capacity of endogenous antioxidant defenses, disrupting intracellular redox homeostasis [33]. While physiological ROS levels are critical for placental development and cellular signaling, excessive ROS leads to accumulation of damaged DNA, misfolded proteins, and lipid peroxides [34,35]. The developing fetus is particularly susceptible due to limited antioxidant capacity and high oxygen demand during rapid growth [11,12].

Maternal insults such as preeclampsia, diabetes, and hypoxia elevate oxidative stress biomarkers, including 8-hydroxydeoxyguanosine (8-OHdG), in both placenta and offspring [36,37]. In the fetal kidney, oxidative stress impairs nephrogenesis, reducing nephron endowment—a key contributor to adult hypertension and chronic kidney disease [38]. It also disrupts nitric oxide (NO) signaling via increased asymmetric dimethylarginine (ADMA), promoting endothelial dysfunction and overactivation of the renin–angiotensin system (RAS), which exacerbates cardiovascular and renal risks later in life [39,40,41]. Mitochondrial dysfunction amplifies this effect, as electron leakage from the respiratory chain increases superoxide production, establishing a “vicious cycle” of oxidative damage [42]. These insights suggest that redox-targeted therapies may hold promise for reprogramming adverse DOHaD-related outcomes in offspring (Figure 1).

### 2.3. Mechanisms of Antioxidant Reprogramming

Within the DOHaD framework, antioxidant reprogramming is predicated on the concept that excessive oxidative stress during critical developmental windows is a central driver of maladaptive programming [43], and that timely perinatal antioxidant interventions can mitigate or reverse these processes before overt disease develops [32,38]. At the mechanistic core of this strategy is the restoration of redox balance and NO bioavailability, exemplified by L-citrulline–mediated reduction of the endogenous NOS inhibitor ADMA, thereby preventing oxidative stress-driven endothelial dysfunction and programmed hypertension [40]. Antioxidant reprogramming further counteracts oxidative stress-induced dysregulation of the RAS by suppressing the classical pro-oxidant, pro-hypertensive axis while favoring activation of the ACE2–angiotensin (1–7)–Mas receptor pathway [41]. In parallel, attenuation of oxidative stress activates redox-sensitive nutrient-sensing pathways, including AMPK/SIRT1/PGC-1α signaling, which supports mitochondrial biogenesis, improves energy homeostasis, and enhances cellular antioxidant capacity in the context of maternal nutritional or metabolic insults [44,45]. Emerging evidence also indicates that antioxidant reprogramming modulates the gut microbiota and its associated metabolites, such as short-chain fatty acids (SCFAs) and hydrogen sulfide (H_2_S), thereby indirectly shaping oxidative stress during development [46,47,48,49]. Collectively, a broad spectrum of antioxidants—including vitamins C and E [50,51], polyphenols [52], resveratrol [53,54], L-citrulline [55], NAC [56], and melatonin—converge on epigenetic and molecular targets during sensitive developmental periods to confer long-term organ resilience and physiological stability. Among these candidates, melatonin has attracted particular attention owing to its potent antioxidative, pleiotropic, and chronobiotic properties [13,14,15,16].

## 3. Melatonin in Pregnancy

### 3.1. Biosynthesis and Metabolism

Melatonin (N-acetyl-5-methoxytryptamine), the “hormone of darkness”, is a versatile indoleamine whose synthesis is predominantly orchestrated in the pinealocytes of the pineal gland under the tight regulation of the hypothalamic suprachiasmatic nucleus (SCN) [57]. The classical biosynthetic pathway initiates with the hydroxylation of the essential amino acid L-tryptophan by tryptophan hydroxylase (TPH) to form 5-hydroxytryptophan (5-HTP), which is subsequently decarboxylated by aromatic L-amino acid decarboxylase (AADC) into serotonin (5-hydroxytryptamine) [58]. The conversion of serotonin to melatonin involves two subsequent steps: acetylation by arylalkylamine N-acetyltransferase (AANAT) to yield N-acetylserotonin (NAS), followed by methylation by acetylserotonin O-methyltransferase (ASMT), formerly known as hydroxyindole O-methyltransferase (HIOMT). AANAT is regarded as the rate-limiting “timezyme” because its activity exhibits a significant 30–70-fold increase during the dark phase, driving the rhythmic nocturnal peak of melatonin [59].

Once synthesized, melatonin is readily released into the systemic circulation and cerebrospinal fluid via passive diffusion due to its high specify and amphiphilicity. In the blood, approximately 70% of melatonin is bound to albumin [60]. Hepatic metabolism serves as the primary route of clearance, where the P450 monooxygenase isoform CYP1A2 hydroxylates melatonin to 6-hydroxymelatonin, which is then conjugated with sulfate to produce 6-sulfatoxymelatonin (aMT6s) for urinary excretion [61]. Beyond indolic metabolism, melatonin is catabolized through a kynuric pathway where the pyrrole ring is cleaved by indoleamine 2,3-dioxygenase to produce N^1^-acetyl-N^2^-formyl-5-methoxykynuramine (AFMK), which is further deformylated into N1-acetyl-5-methoxykynuramine (AMK). These metabolites are critical components of an “antioxidant cascade”, as they retain potent radical-scavenging properties, allowing a single melatonin molecule to neutralize up to ten reactive species [57].

### 3.2. The Placenta as an Extrapineal Source

A paradigm-shifting realization in reproductive medicine is that more than 95% of the body’s total melatonin production is of extrapineal origin [62], with the placenta serving as a major biosynthetic site during gestation [63]. Unlike the pineal gland, the placenta synthesizes melatonin in a non-circadian, constant manner. Both mononuclear villous cytotrophoblasts and multinucleated syncytiotrophoblasts possess the enzymatic machinery (AANAT and ASMT) necessary for de novo melatonin synthesis [17].

Maternal serum melatonin levels escalate significantly during pregnancy, reaching peak concentrations at term and plummeting abruptly postpartum, which reflects the significant contribution of the fetoplacental unit to the maternal pool [17]. Placental melatonin operates via autocrine and paracrine mechanisms to maintain organostasis. Specifically, locally produced melatonin acts as a “watchdog” for trophoblast homeostasis, inhibiting apoptosis of the cytotrophoblast while promoting its syncytialization into the syncytiotrophoblast. This local production provides “on-site protection” against the excessive production of ROS associated with placental senescence and complications like preeclampsia [20].

### 3.3. Melatonin Receptor Signaling and Placental Barrier

The multifaceted biofunctions of melatonin are mediated by three primary targets at the maternal-fetal interface. MT1 (Mel1a, encoded by chromosome 4) and MT2 (Mel1b, encoded by chromosome 11) are 7-transmembrane G-protein coupled receptors (GPCRs) widely expressed in fetal tissues, the pituitary gland, and the placenta [64]. MT1 typically couples with Gi proteins to inhibit adenylyl cyclase/cAMP/PKA signaling, while MT2 is involved in phosphoinositol hydrolysis and cGMP inhibition. A third binding site, MT3, has been pharmacologically characterized as the cytosolic enzyme quinone reductase 2 (NQO2), which functions in detoxification and redox protection [65].

**Figure 2:**
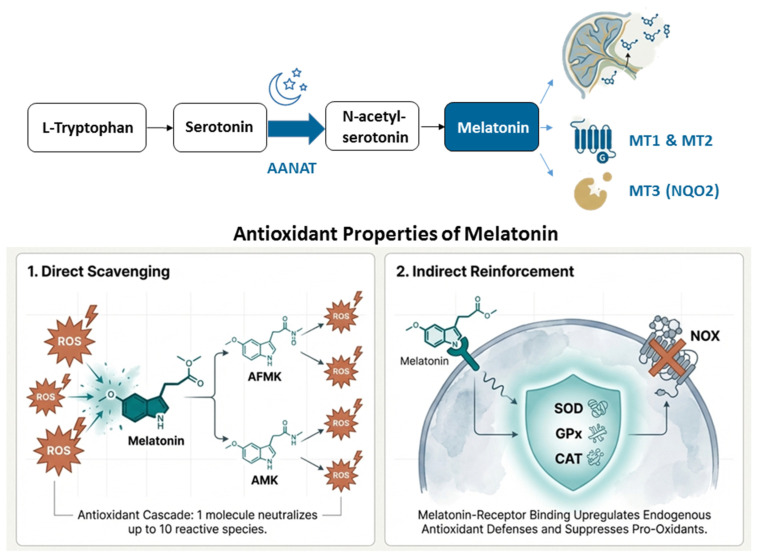
Antioxidant actions of melatonin during pregnancy.

Melatonin’s unique amphiphilic and lipophilic properties allow it to freely and rapidly cross all biological barriers, including the placental barrier and the fetal blood–brain barrier, without being significantly metabolized. Since the fetal pineal gland does not achieve rhythmic secretion until 3–5 months postnatally, the fetus depends entirely on the maternal melatonin rhythm to perceive photoperiodic signals [66]. Maternal melatonin acts on the fetal suprachiasmatic nucleus to entrain the developing circadian system, a process essential for the programming of postnatal behavior and physiological timing [20]. The role of melatonin during pregnancy is summarized in Figure 2.

## 4. Reprogramming Effects of Melatonin on Offspring Organ Systems

Melatonin, capable of crossing placental and blood–brain barriers, delivers essential chronobiotic and antioxidant signals during pregnancy [20]. While some human studies have explored its use during pregnancy and lactation, comprehensive reviews reveal a lack of focused long-term offspring assessments [21,22]. Table 1 summarizes studies documenting melatonin’s reprogramming effects in rodent models of CKM syndrome [67,68,69,70,71,72,73,74,75]. As a reprogramming agent, it redirects therapy to the perinatal period, preventing or reversing maladaptive programming across multiple organ systems.

### 4.1. Kidney Programming

The kidney is highly sensitive to the intrauterine environment, and kidney programming is a key determinant of lifelong susceptibility to hypertension and CKD [76]. Nephron endowment critically influences this risk, as a reduced nephron number limits renal reserve and necessitates compensatory hyperfiltration to maintain GFR, thereby increasing intraglomerular pressure and predisposing to hypertension, glomerulosclerosis, and CKD—effects that are further exacerbated by later-life ‘second hits’ [77,78]. Prenatal dexamethasone exposure has been shown to reduce nephron number, resulting in glomerular hyperfiltration and subsequent kidney injury, whereas maternal melatonin supplementation prevents this loss of nephron endowment and preserves renal structural integrity [68].

The reprogramming effects of melatonin are closely linked to modulation of the intrarenal renin–angiotensin system (RAS). Aberrant activation of the classical RAS axis (ACE/Ang II/AT1R) is a hallmark of programmed hypertension [41], while melatonin counteracts this imbalance by enhancing the protective non-classical axis (ACE2/Ang 1–7/Mas receptor). In experimental models of maternal caloric restriction [67], high-fructose intake [74], and continuous light exposure [70], perinatal melatonin administration effectively suppressed classical RAS activation and prevented the development of offspring hypertension.

Consistent with these functional effects, whole-genome RNA next-generation sequencing revealed that maternal melatonin therapy induces sustained alterations in the renal transcriptome [79]. In one-week-old offspring, melatonin upregulated 455 genes associated with critical biological pathways, including AMPK signaling, TGF-β signaling, and tryptophan metabolism, supporting the concept that melatonin acts as a genomic inducer during critical windows of kidney development.

### 4.2. Cardiovascular Programming

CKM syndrome critically depends on vascular integrity and myocardial resilience. Melatonin functions as a ‘cardiovascular guardian’ through multiple complementary mechanisms. First, melatonin restores nitric oxide (NO) bioavailability, which is essential for vascular tone and blood pressure regulation. Oxidative stress increases asymmetric dimethylarginine (ADMA), an endogenous inhibitor of nitric oxide synthase, thereby disrupting NO–ROS balance [80,81]. Maternal melatonin therapy prevents hypertension by reducing ADMA levels and enhancing renal NO availability, effectively reestablishing redox homeostasis [67]. Second, in models of maternal hypoxia, melatonin mitigates cardiac wall thinning and increases myocardial eNOS protein expression, thereby strengthening cardiac resilience to subsequent stressors [71]. Moreover, offspring born to diabetic mothers exhibit heightened susceptibility to myocardial ischemia–reperfusion injury; maternal melatonin supplementation significantly improves ischemic tolerance by reducing infarct size, cardiac dysfunction, and myocardial apoptosis in these offspring [72].

### 4.3. Metabolic Programming

Metabolic programming within the CKM framework primarily determines future susceptibility to obesity, diabetes, and dyslipidemia [82,83]. Melatonin plays an integral role in regulating glucose homeostasis through interactions with glucose and insulin signaling pathways [84]. Maternal nutritional imbalance is known to program adult glucose intolerance and insulin resistance [73,74]; however, melatonin supplementation during gestation and lactation has been shown to ameliorate these adverse metabolic phenotypes by preserving pancreatic β-cell function and enhancing peripheral insulin sensitivity.

In addition, maternal overnutrition, particularly high-fat diets, programs offspring obesity and hyperlipidemia [73]. Maternal melatonin therapy robustly suppresses excessive body weight gain and visceral adiposity in these models. Given that hepatic steatosis is a central component of CKM syndrome, melatonin also confers protection against diet-induced liver steatosis in offspring, an effect associated with reduced hepatic oxidative stress and reversal of leptin gene hypermethylation [73]. Similarly, in maternal high-fructose diet models, melatonin prevents the development of hypertension and dyslipidemia through activation of AMPK signaling [74].

### 4.4. Mechanisms of Melatonin Reprogramming

**Figure 3:**
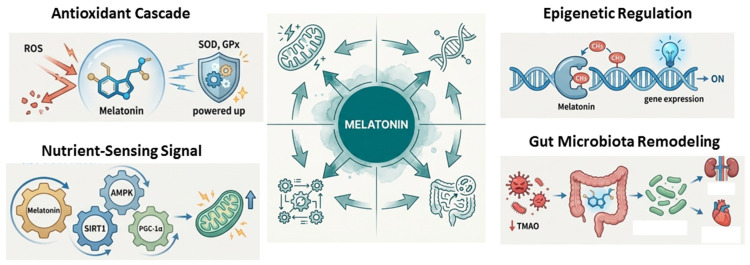
Reprogramming effects of melatonin.

The efficacy of melatonin as a reprogramming agent in CKM syndrome derives from its unique ability to coordinate multiple protective pathways across organs, including the kidney, heart, liver, and vasculature [13,19,79]. By simultaneously modulating redox homeostasis, mitochondrial integrity, epigenetic regulation, nutrient-sensing signaling, and gut microbiota composition, melatonin provides a comprehensive defense against the multifactorial insults imposed by developmental programming. This multi-targeted action distinguishes melatonin from conventional single-target antioxidants and underlies its potent reprogramming capacity (Figure 3).

#### 4.4.1. Antioxidant Cascade

Melatonin and its metabolites, such as AFMK and AMK, operate as a sequential radical-scavenging cascade capable of neutralizing up to ten reactive species per molecule [85]. These include highly cytotoxic radicals such as hydroxyl radicals (·OH), peroxynitrite (ONOO^−^), singlet oxygen, and superoxide anions. Direct scavenging prevents lipid peroxidation, protein oxidation, and DNA damage at the cellular level. Beyond direct action, melatonin exerts profound indirect antioxidant effects by enhancing the expression and enzymatic activity of endogenous antioxidants, including superoxide dismutase (SOD), glutathione peroxidase (GPx), and catalase (CAT), while downregulating pro-oxidant enzymes such as NADPH oxidase (NOX) and inducible nitric oxide synthase (iNOS) [86]. This rebalancing of the redox state restores NO bioavailability by lowering levels of ADMA [81], which is critical for maintaining vascular tone and cellular signaling.

Melatonin also stabilizes mitochondrial function through the reduction of electron leakage from complexes I and IV of the electron transport chain, a mechanism termed “radical avoidance” [87]. By preventing ROS overproduction at its primary source, melatonin preserves mitochondrial membrane potential, inhibits cytochrome c release, and prevents apoptosis induced by oxidative stress. These combined actions protect cellular bioenergetics and tissue integrity, particularly in organs vulnerable to CKM programming-induced oxidative damage.

#### 4.4.2. Epigenetic Regulation

Melatonin functions as an epigenetic modulator, inhibiting DNA methyltransferases (DNMTs) and histone deacetylases (HDACs) [18,79]. Through these actions, melatonin reverses aberrant promoter hypermethylation and histone modifications that are commonly induced by prenatal stress [88,89,90]. In experimental models, melatonin restores the expression of critical developmental genes, including reelin and leptin, highlighting its role in correcting maladaptive epigenetic programming [89,90]. Such modifications are particularly relevant for organ systems where early life stress leads to long-term dysfunction, as epigenetic reprogramming by melatonin can normalize gene networks controlling metabolism, vascular function, and cellular stress responses.

#### 4.4.3. Nutrient-Sensing Signaling

Melatonin activates key nutrient-sensing pathways, including AMPK, SIRT1, and PGC-1α, which regulate mitochondrial biogenesis, energy expenditure, and metabolic homeostasis [91,92]. Activation of these pathways counteracts metabolic programming induced by maternal imbalanced nutrition [73,74]. By promoting mitochondrial function and enhancing cellular energy efficiency, melatonin mitigates the downstream consequences of impaired nutrient signaling [92], including insulin resistance, dyslipidemia, and hepatic steatosis, all hallmark features of CKM syndrome [13,32].

#### 4.4.4. Gut Microbiota Remodeling

Accumulating evidence supports a contributory role of melatonin in mitigating CKM syndrome through modulation of the gut microbiota, a central regulator of the gut–kidney and gut–heart axes [93,94,95,96]. Melatonin administration has been shown to enhance microbial α-diversity and enrich beneficial taxa, including *Roseburia*, thereby improving host metabolite profiles such as the plasma trimethylamine-N-oxide-to-trimethylamine ratio and reducing the systemic burden of uremic toxins and cardiovascular risk [97]. Beyond compositional changes, melatonin influences microbial richness and temporal dynamics, with downstream effects on intestinal permeability and host–microbe metabolic interactions. Through these mechanisms, melatonin helps establish a favorable cellular and metabolic milieu that supports cardiovascular development during early life [94]. Collectively, sustained remodeling of the gut microbiota by melatonin confers long-term benefits on host metabolism [93,94], inflammatory tone [95], and vascular function [96], underscoring its multi-organ protective potential within the CKM framework.

#### 4.4.5. Translational Considerations

Collectively, evidence derived from animal models provides a strong mechanistic rationale for melatonin as a reprogramming agent with the potential to attenuate the lifelong burden of CKM syndrome. By simultaneously modulating oxidative stress, renal RAS activation, epigenetic regulation, nutrient-sensing pathways, and the gut microbiota, melatonin confers systemic protective actions that are difficult to achieve with conventional single-target strategies. Despite this compelling preclinical foundation, a substantial translational gap remains [98]. In the rat models summarized in Table 1, maternal melatonin administration at doses ranging from 0.05 to 10 mg/kg/day consistently protects offspring from CKM-related phenotypes. Importantly, melatonin demonstrates an exceptionally favorable safety profile in rodents, with a reported intraperitoneal LD_50_ of 1168 mg/kg and no defined lethal dose following oral administration up to 3200 mg/kg [99]. Based on body surface area conversion, these data correspond to estimated human-equivalent doses of approximately 40–100 mg/day [100,101]. Nevertheless, rigorous studies are still required to establish standardized dosing regimens, define critical windows of intervention, and systematically assess the long-term safety and developmental consequences of high-dose melatonin exposure during pregnancy and across the lifespan of offspring.

## 5. Clinical Implications of Melatonin in Compromised Pregnancies

Although direct human evidence linking maternal melatonin supplementation to reduced offspring CKM syndrome remains limited, melatonin has already been explored clinically to improve adverse pregnancy outcomes. Gestation represents a period of exceptional developmental plasticity, during which environmental insults can permanently redirect fetal organ development. When the maternal–placental–fetal unit is compromised, oxidative stress emerges as a central driver of maladaptive programming. Owing to its unique sequential radical-scavenging antioxidant cascade and its ability to freely cross biological barriers, melatonin provides coordinated, pleiotropic protective functions against key pathophysiological components underlying CKM syndrome.

### 5.1. Preeclampsia: Addressing Placental Insufficiency and Systemic Oxidative Stress

Preeclampsia is a human pregnancy–specific, multisystem disorder defined by the de novo onset of hypertension in conjunction with maternal end-organ dysfunction [102]. Its etiopathogenesis is widely attributed to aberrant placentation, characterized by insufficient spiral artery remodeling that culminates in a high-resistance uteroplacental circulation and chronic ischemia–reperfusion stress. This maladaptive intrauterine milieu precipitates excessive reactive oxygen species generation and facilitates the systemic release of antiangiogenic mediators, notably soluble fms-like tyrosine kinase-1, into the maternal compartment [102]. Converging clinical and experimental evidence demonstrates a profound disruption of the placental melatonergic system in severe preeclampsia, reflected by markedly attenuated nocturnal circulating melatonin levels alongside suppressed expression of key synthetic enzymes (AANAT and ASMT) and receptors (MT1 and MT2) within placental tissue [103,104]. The resultant melatonin deficiency compromises local cytoprotective and antioxidant defenses, promoting cytotrophoblast apoptosis and destabilizing the syncytiotrophoblast barrier. From a translational standpoint, the PAMPR phase I pilot study established that high-dose melatonin supplementation (30 mg/day) is well tolerated in early onset preeclampsia and extends the diagnosis-to-delivery interval by approximately six days, a clinically meaningful window for fetal maturation [105]. Nonetheless, optimal dosing, timing, and long-term maternal–offspring safety remain undetermined. Building on this foundation, the MELPOP trial evaluates prophylactic high-dose melatonin in moderate- and high-risk pregnancies, with emerging data indicating maternal–fetal safety, prolongation of gestational latency following diagnosis, and a reduced requirement for escalation of antihypertensive therapy. Collectively, these findings support the concept that exogenous melatonin may counteract defective placental–uterine interface establishment by virtue of its potent antioxidant and endothelial-protective actions, functioning as a molecular “watchdog” of trophoblast homeostasis and attenuating oxidative programming processes that predispose offspring to adverse CKM-related phenotypes [106].

### 5.2. Intrauterine Growth Restriction: Restoring Redox Balance and Improving Placental Perfusion

Intrauterine growth restriction (IUGR), most commonly resulting from placental insufficiency [107], reflects a state of fetal redox imbalance that impairs attainment of the genetically programmed growth trajectory and constitutes a well-established developmental precursor of adult-onset CKM syndrome [108,109]. Within this adverse intrauterine context, the fetus undergoes adaptive “thrifty” programming that preferentially preserves cerebral growth while constraining nephron endowment and long-term metabolic reserve. Accumulating evidence indicates that IUGR pregnancies are characterized by a profound disruption of the placental melatonergic axis, with significantly reduced melatonin concentrations and diminished MT receptor expression detected in both maternal and umbilical cord blood [110,111,112]. This deficiency contributes to a pro-inflammatory, pro-oxidative placental microenvironment, marked by enhanced lipid peroxidation and elevated levels of malondialdehyde, indicative of impaired redox homeostasis [113].

Clinical exploration of melatonin in IUGR has therefore centered on its capacity to restore redox balance and stabilize placental function within the maternal–placental–fetal unit. In a seminal phase I study, Miller et al. demonstrated that oral melatonin administration at 8 mg/day significantly attenuated placental malondialdehyde concentrations without eliciting adverse maternal or fetal outcomes, despite the absence of a statistically significant increase in birth weight [113]. These studies are promising but limited in scale, duration, and generalizability. These reassuring safety and mechanistic signals have catalyzed larger interventional trials, including NCT05651347, which are currently assessing higher-dose regimens (30 mg/day) aimed at mitigating neurodevelopmental injury and cerebral vulnerability in severe preterm IUGR [114]. Consistent with these interventional efforts, observational data further reveal that umbilical cord melatonin and placental growth factor levels are markedly depleted in IUGR-complicated pregnancies relative to normally grown fetuses, reinforcing the concept that melatonin insufficiency constitutes a central pathogenic node linking placental oxidative stress, impaired perfusion, and maladaptive developmental programming.

### 5.3. Gestational Diabetes: Genetic Variants of MTNR1B and the Regulation of Glycemic Control

Gestational diabetes is a disorder of carbohydrate metabolism that increases the lifetime risk of type 2 diabetes and metabolic syndrome in both mothers and offspring [115]. Genetic studies have established a robust association between polymorphisms in the melatonin receptor 1B gene and susceptibility to GDM, particularly the rs10830963 G allele, which is linked to impaired β-cell function and elevated fasting glucose levels [116,117]. Melatonin plays a dual role in glucose homeostasis: it enhances insulin sensitivity and β-cell survival through antioxidant and mitochondrial effects, while physiologically suppressing nocturnal insulin secretion to prevent hypoglycemia. In GDM, reduced melatonin levels correlate with worsening glycemic control [118]. In rodent models, supplementation rescues the diabetic phenotype via activation of the AMPK/SIRT1 pathway, promoting mitochondrial biogenesis and metabolic flexibility [119]. However, translation to humans is limited by differences in physiology, dose equivalence, and gestational timing. Human studies in hyperglycemic pregnancies suggest that melatonin at 10 mg/day may outperform quercetin in improving maternal glycemic control and reducing neonatal complications such as macrosomia and respiratory distress [22], though larger and longer-term trials are needed to confirm efficacy and safety.

### 5.4. Current Human Trials and the Translational Gap

The clinical trial portfolio for melatonin has expanded substantially, with more than 600 registered studies worldwide; however, only a small fraction—approximately 40 trials—specifically address pregnancy-related indications, and even fewer incorporate assessments of long-term offspring outcomes [114]. This imbalance underscores a critical disconnect between the growing enthusiasm for melatonin use and the paucity of rigorous developmental safety data. Notably, a U.S. national survey reports that nearly 4% of pregnant women already self-administer melatonin as a dietary supplement [120], highlighting a widening gap between real-world exposure and evidence-based guidance. However, most evidence derives from preclinical rodent studies, which have inherent limitations including species-specific differences in metabolism [121], placentation [122], gestational period [123], and developmental timing [124], as well as heterogeneity in dosing and experimental endpoints [125]. Consequently, direct extrapolation to human pregnancy remains challenging, underscoring the need for proof-of-concept validation and early phase clinical trials. Despite this increasing utilization, robust longitudinal data evaluating maternal–fetal safety and postnatal cardiometabolic trajectories remain largely absent [22,126,127,128,129,130].

A recent scoping review by Vine et al. (2022) characterized melatonin as “probably safe” in humans, based on clinical trials employing doses ranging from 8 to 30 mg/day without major adverse events [22]. Nonetheless, the majority of preclinical studies suggest that the human-equivalent doses required to achieve meaningful antioxidant reprogramming effects may lie in the range of 40–100 mg/day [100,101]—substantially higher than both over-the-counter formulations and doses currently tested in pregnancy trials. Notably, melatonin may exert pro-oxidant effects at supraphysiological concentrations (1–10 mM) [131], underscoring its role as a context-dependent regulator rather than a classical hormone and highlighting the critical need to define safe upper dosing limits in humans. These considerations emphasize the limitations of directly extrapolating rodent data to human pregnancy and the necessity of careful dose selection.

Addressing these translational gaps will require carefully designed, multicenter randomized controlled trials that explicitly interrogate dose–response relationships, timing, and duration of supplementation, while incorporating extended longitudinal follow-up of exposed offspring. Integration into preventive strategies should also consider biomarker-based risk stratification to identify pregnancies most likely to benefit. Such trials are essential to determine whether perinatal melatonin supplementation can move beyond short-term biochemical modulation and genuinely alter the developmental trajectory underlying CKM trait emergence.

## 6. Materials and Methods

Owing to the conceptual complexity and methodological diversity of the available literature, we employed a narrative review approach rather than a systematic or scoping review, allowing for an integrative synthesis of emerging insights across redox signaling, developmental biology, clinical research, and animal studies. A comprehensive literature search was conducted using MEDLINE, Embase, and the Cochrane Library to identify English-language studies. The search strategy included terms related to melatonin, oxidative stress, DOHaD, and CKM syndrome, specifically: “melatonin”, “pineal hormone”, “circadian rhythm”, “nitric oxide”, “oxidative stress”, “reactive oxygen species”, “reactive nitrogen species”, “free radicals”, “developmental programming”, “life course theory”, “DOHaD”, “offspring”, “progeny”, “mother”, “prenatal”, “pregnancy”, “reprogramming”, “metabolic syndrome”, “hypertension”, “dyslipidemia”, “hyperlipidemia”, “obesity”, “diabetes”, “insulin resistance”, “hyperglycemia”, “liver steatosis”, “chronic kidney disease”, “cardiovascular disease”, “atherosclerosis”, “heart failure”, and “cardiorenal syndrome”. Reference lists of retrieved articles were also examined to capture additional relevant studies. The search covered publications from January 2000 through December 2025.

## 7. Future Perspectives and Conclusions

Melatonin is increasingly recognized as a pleiotropic regulator of biological homeostasis, with its antioxidant capacity emerging as a unifying mechanism across the life course. Beyond its conventional role as a circadian signal, melatonin stabilizes redox balance through potent cytoprotective actions at both systemic and cellular levels.

Although melatonin is a potent redox modulator capable of preventing CKM syndrome, not all antioxidants provide equivalent protection. Our previous review shows that polyphenols can also mitigate CKM risk [132]. Both melatonin and polyphenols restore redox balance during critical developmental windows, activate nutrient-sensing pathways (AMPK/SIRT1), modulate the gut microbiota, rebalance the RAS, and influence epigenetic mechanisms. However, melatonin is distinctive as a “mitochondrial watchdog”, with a sequential antioxidant cascade that enables a single molecule and its metabolites to neutralize multiple reactive species, deliver chronobiotic signals essential for fetal circadian entrainment, stabilize mitochondrial electron flow (“radical avoidance”), and freely cross biological barriers including the placenta. In contrast, polyphenols, while exhibiting antioxidant and anti-inflammatory actions, are limited by low bioavailability, variable pharmacokinetics, and potential interference with digestive or hormonal processes at high doses, emphasizing that antioxidant effects are context-dependent and molecule-specific.

By directly scavenging reactive oxygen and nitrogen species and engaging downstream antioxidant cascades, melatonin extends its protective influence beyond its immediate bioavailability, thereby preserving mitochondrial function and cellular integrity under conditions of metabolic and vascular stress. From a holistic perspective, melatonin facilitates allostatic adaptation in both the maternal–fetal unit and the aging organism, whereas disruption of its rhythmic secretion amplifies allostatic load and accelerates chronic disease progression. Acting as an antioxidant sentinel, melatonin and its bioactive metabolites integrate redox control with epigenetic regulation, modulation of nutrient-sensing pathways, and reshaping of the gut microbiota—processes that collectively converge on the developmental and lifelong trajectories underlying CKM-related phenotypes.

Within the DOHaD framework, the perinatal period represents a uniquely sensitive window during which physiological systems are simultaneously vulnerable to insult and amenable to therapeutic reprogramming. Substantial preclinical evidence supports this concept, demonstrating that perinatal melatonin supplementation can prevent programmed hypertension, metabolic dysfunction, cardiovascular disease, and hepatic steatosis in offspring exposed to diverse prenatal challenges. Despite these promising findings, clinical translation remains limited by unresolved questions regarding optimal timing, duration, and dosing strategies. Early human studies indicate that pharmacological doses are well tolerated within the maternal–fetal unit; however, definitive long-term safety and efficacy data are still lacking. Addressing these gaps will require carefully designed studies to determine whether intervention should be restricted to late gestation, extended through lactation, or tailored to specific developmental endpoints.

In conclusion, melatonin should no longer be regarded solely as a hypnotic agent but rather as a central stabilizer of temporal and redox homeostasis—a biological integrator aligning internal physiology with environmental and metabolic cues. Nevertheless, extrapolation from animal studies to human prevention strategies should be undertaken cautiously until robust, long-term clinical data are available. Although clinical translation is still in its early stages, the strategic incorporation of melatonin into prenatal and pediatric care paradigms holds the potential to fundamentally reshape preventive approaches to hypertension and metabolic disease. Progress in this field will depend on a decisive transition from anecdotal use and small-scale trials to rigorously designed, long-term human studies capable of validating melatonin as a cornerstone intervention for reducing the lifelong burden of CKM syndrome.

## Figures and Tables

**Table 1:** Summary of Melatonin Reprogramming in CKM Syndrome Models.

CKM Component	Animal Model (Maternal Insult)	Melatonin Dose/Timing	Reprogramming Effects & Mechanisms	Ref.
Kidney/HTN	50% Caloric Restriction (Malnutrition)	0.01% in water (G/L)	Prevents HTN; ↓ ADMA; ↑ Renal NO; ↑ ACE2 expression	[67]
Kidney/HTN	Prenatal Dexamethasone (Drug exposure)	0.01% in water (G/L)	Prevents HTN; ↑ Nephron endowment	[68]
Kidney/HTN	L-NAME (Preeclampsia)	0.01% in water (G/L)	Prevents HTN; Restores renal transcriptome and H_2_S pathway	[69]
Kidney/HTN	Continuous light exposure (Chronodisruption)	0.01% in water (G/L)	Prevents HTN; ↓ Renal sodium transporter expression	[70]
Cardiovascular	Maternal Hypoxia (Pregnancy complication)	0.05 mg/kg p.o. (G)	Improves myocardial resilience; ↑ Cardiac eNOS protein	[71]
Cardiovascular	Maternal Diabetes (Maternal illness)	0.01% in water (G/L)	Improves myocardial ischemic tolerance; ↓ Infarct size	[72]
Metabolic	High-Fat Diet (Maternal overnutrition)	5 mg/kg i.p. (G/L)	Prevents obesity, hyperglycemia, and liver steatosis	[73]
Metabolic	60% High-Fructose Diet (Maternal overnutrition)	0.01% in water (G/L)	Prevents HTN and dyslipidemia; ↑ AMPK activity	[74]
Metabolic	Maternal pinealectomy (Chronodisruption)	0.5 mg/kg p.o. (G/L)	Restores daily rhythm of energy metabolism	[75]

HTN = hypertension; G = gestation; L = lactation; p.o. = per oral; i.p. = intraperitoneal; L-NAME = N^G^-nitro-l-arginine methyl ester; ADMA = asymmetric dimethylarginine; ACE2 = angiotensin converting enzyme 2; eNOS = endothelial nitric oxide synthase.

## Data Availability

No new data were created or analyzed in this study. Data sharing is not applicable to this review article.

## References

[1] Ferrari A.J., Santomauro D.F., Aali A., Abate Y.H., Abbafati C., Abbastabar H. (2024). GBD 2021 Diseases and Injuries Collaborators. Global incidence, prevalence, years lived with disability (YLDs), disability-adjusted life-years (DALYs), and healthy life expectancy (HALE) for 371 diseases and injuries in 204 countries and territories and 811 subnational locations, 1990–2021: A systematic analysis for the Global Burden of Disease Study 2021. Lancet.

[2] Ndumele C.E., Neeland I.J., Tuttle K.R., Chow S.L., Mathew R.O., Khan S.S., Coresh J., Baker-Smith C.M., Carnethon M.R., Després J.P. (2023). A Synopsis of the Evidence for the Science and Clinical Management of Cardiovascular-Kidney-Metabolic (CKM) Syndrome: A Scientific Statement from the American Heart Association. Circulation.

[3] Jaradat J.H., Nashwan A.J. (2023). Cardiovascular-kidney-metabolic syndrome: Understanding the interconnections and the need for holistic intervention. J. Med. Surg. Public Health.

[4] Aggarwal R., Ostrominski J.W., Vaduganathan M. (2024). Prevalence of Cardiovascular-Kidney-Metabolic Syndrome Stages in US Adults, 2011–2020. JAMA.

[5] Khan S.S., Coresh J., Pencina M.J., Ndumele C.E., Rangaswami J., Chow S.L., Palaniappan L.P., Sperling L.S., Virani S.S., Ho J.E. (2023). Novel Prediction Equations for Absolute Risk Assessment of Total Cardiovascular Disease Incorporating Cardiovascular-Kidney-Metabolic Health: A Scientific Statement from the American Heart Association. Circulation.

[6] Fleming T.P., Velazquez M.A., Eckert J.J. (2015). Embryos, DOHaD and David Barker. J. Dev. Orig. Health Dis..

[7] Dev A., Patra R., Kale S.A., James Marianadin L.P., Mohanty S.S., Benny J. (2025). From Womb to Tomb: A Life-Course Perspective on the Origins of Non-communicable Diseases. Cureus.

[8] Hanson M., Gluckman P. (2016). Commentary: Developing the future: Life course epidemiology, DOHaD and evolutionary medicine. Int. J. Epidemiol..

[9] Padmanabhan V., Cardoso R.C., Puttabyatappa M. (2016). Developmental Programming, a Pathway to Disease. Endocrinology.

[10] Tain Y.L., Joles J.A. (2015). Reprogramming: A Preventive Strategy in Hypertension Focusing on the Kidney. Int. J. Mol. Sci..

[11] Thompson L.P., Al-Hasan Y. (2012). Impact of oxidative stress in fetal programming. J. Pregnancy.

[12] Dennery P.A. (2010). Oxidative stress in development: Nature or nurture?. Free Radic. Biol. Med..

[13] Tain Y.L., Hsu C.N. (2024). Melatonin Use during Pregnancy and Lactation Complicated by Oxidative Stress: Focus on Offspring’s Cardiovascular-Kidney-Metabolic Health in Animal Models. Antioxidants.

[14] Reiter R.J., Mayo J.C., Tan D.X., Sainz R.M., Alatorre-Jimenez M., Qin L. (2016). Melatonin as an Antioxidant: Under Promises but over Delivers. J. Pineal Res..

[15] Ghorbaninejad P., Sheikhhossein F., Djafari F., Tijani A.J., Mohammadpour S., Shab-Bidar S. (2020). Effects of melatonin supplementation on oxidative stress: A systematic review and meta-analysis of randomized controlled trials. Horm. Mol. Biol. Clin. Investig..

[16] Tordjman S., Chokron S., Delorme R., Charrier A., Bellissant E., Jaafari N., Fougerou C. (2017). Melatonin: Pharmacology, Functions and Therapeutic Benefits. Curr. Neuropharmacol..

[17] Tamura H., Nakamura Y., Terron M.P., Flores L.J., Manchester L.C., Tan D.X., Sugino N., Reiter R.J. (2008). Melatonin and pregnancy in the human. Reprod. Toxicol..

[18] Korkmaz A., Reiter R.J. (2008). Epigenetic regulation: A new research area for melatonin?. J. Pineal Res..

[19] Tain Y.L., Huang L.T., Hsu C.N. (2017). Developmental Programming of Adult Disease: Reprogramming by Melatonin?. Int. J. Mol. Sci..

[20] Gomes P.R.L., Motta-Teixeira L.C., Gallo C.C., Carmo Buonfiglio D.D., Camargo L.S., Quintela T., Reiter R.J., Amaral F.G.D., Cipolla-Neto J. (2021). Maternal pineal melatonin in gestation and lactation physiology, and in fetal development and programming. Gen. Comp. Endocrinol..

[21] Aversa S., Pellegrino S., Barberi I., Reiter R.J., Gitto E. (2012). Potential utility of melatonin as an antioxidant during pregnancy and in the perinatal period. J. Matern. Fetal Neonatal Med..

[22] Vine T., Brown G.M., Frey B.N. (2022). Melatonin use during pregnancy and lactation: A scoping review of human studies. Braz. J. Psychiatry.

[23] Barker D.J., Eriksson J.G., Forsen T., Osmond C. (2002). Fetal origins of adult disease: Strength of effects and biological basis. Int. J. Epidemiol..

[24] Roseboom T., de Rooij S., Painter R. (2006). The Dutch famine and its long-term consequences for adult health. Early Hum. Dev..

[25] Sun C., Burgner D.P., Ponsonby A.L., Saffery R., Huang R.C., Vuillermin P.J., Cheung M., Craig J.M. (2013). Effects of early-life environment and epigenetics on cardiovascular disease risk in children: Highlighting the role of twin studies. Pediatr. Res..

[26] Oken E., Huh S.Y., Taveras E.M., Rich-Edwards J.W., Gillman M.W. (2005). Associations of maternal prenatal smoking with child adiposity and blood pressure. Obes. Res..

[27] Fraser A., Nelson S.M., Macdonald-Wallis C., Sattar N., Lawlor D.A. (2013). Hypertensive disorders of pregnancy and cardiometabolic health in adolescent offspring. Hypertension.

[28] Hsu C.N., Tain Y.L. (2021). Adverse Impact of Environmental Chemicals on Developmental Origins of Kidney Disease and Hypertension. Front. Endocrinol..

[29] Moon J.H., Jang H.C. (2022). Gestational Diabetes Mellitus: Diagnostic Approaches and Maternal-Offspring Complications. Diabetes Metab. J..

[30] Schreuder M.F., Bueters R.R., Huigen M.C., Russel F.G., Masereeuw R., van den Heuvel L.P. (2011). Effect of drugs on renal development. Clin. J. Am. Soc. Nephrol..

[31] Hsu C.N., Tain Y.L. (2020). Light and Circadian Signaling Pathway in Pregnancy: Programming of Adult Health and Disease. Int. J. Mol. Sci..

[32] Tain Y.L., Hsu C.N. (2022). Metabolic Syndrome Programming and Reprogramming: Mechanistic Aspects of Oxidative Stress. Antioxidants.

[33] Sies H. (2015). Oxidative stress: A concept in redox biology and medicine. Redox Biol..

[34] Hussain T., Murtaza G., Metwally E., Kalhoro D.H., Kalhoro M.S., Rahu B.A., Sahito R.G.A., Yin Y., Yang H., Chughtai M.I. (2021). The Role of Oxidative Stress and Antioxidant Balance in Pregnancy. Mediat. Inflamm..

[35] Ibrahim A., Khoo M.I., Ismail E.H.E., Hussain N.H.N., Zin A.A.M., Noordin L., Abdullah S., Mahdy Z.A., Lah N.A.Z.N. (2024). Oxidative stress biomarkers in pregnancy: A systematic review. Reprod. Biol. Endocrinol..

[36] Taysi S., Tascan A.S., Ugur M.G., Demir M. (2019). Radicals, Oxidative/Nitrosative Stress and Preeclampsia. Mini. Rev. Med. Chem..

[37] Myatt L. (2010). Review: Reactive oxygen and nitrogen species and functional adaptation of the placenta. Placenta.

[38] Hsu C.N., Tain Y.L. (2020). Developmental Origins of Kidney Disease: Why Oxidative Stress Matters?. Antioxidants.

[39] Wilcox C.S. (2005). Oxidative stress and nitric oxide deficiency in the kidney: A critical link to hypertension?. Am. J. Physiol. Regul. Integr. Comp. Physiol..

[40] Tain Y.L., Hsu C.N. (2023). The NOS/NO System in Renal Programming and Reprogramming. Antioxidants.

[41] Hsu C.N., Tain Y.L. (2021). Targeting the Renin-Angiotensin-Aldosterone System to Prevent Hypertension and Kidney Disease of Developmental Origins. Int. J. Mol. Sci..

[42] Lu L., Huang X., Shi Y., Jiang Y., Han Y., Zhang Y. (2025). Mitochondrial dysfunction in pregnancy loss: A review. Mol. Cell. Biochem..

[43] Pisoschi A.M., Pop A. (2015). The role of antioxidants in the chemistry of oxidative stress: A review. Eur. J. Med. Chem..

[44] Jansson T., Powell T.L. (2013). Role of placental nutrient sensing in developmental programming. Clin. Obstet. Gynecol..

[45] Efeyan A., Comb W.C., Sabatini D.M. (2015). Nutrient-sensing mechanisms and pathways. Nature.

[46] Magliocca G., Mone P., Di Iorio B.R., Heidland A., Marzocco S. (2022). Short-Chain Fatty Acids in Chronic Kidney Disease: Focus on Inflammation and Oxidative Stress Regulation. Int. J. Mol. Sci..

[47] Hsu C.N., Lin Y.J., Hou C.Y., Chen Y.W., Chang-Chien G.P., Lin S.F., Tain Y.L. (2025). Antioxidants, Gut Microbiota, and Cardiovascular Programming: Unraveling a Triad of Early-Life Interactions. Antioxidants.

[48] Scammahorn J.J., Nguyen I.T.N., Bos E.M., Van Goor H., Joles J.A. (2021). Fighting Oxidative Stress with Sulfur: Hydrogen Sulfide in the Renal and Cardiovascular Systems. Antioxidants.

[49] Hsu C.N., Lin Y.J., Hou C.Y., Chen Y.W., Tain Y.L. (2025). Early-Life Hydrogen Sulfide Signaling as a Target for Cardiovascular-Kidney-Metabolic Syndrome Reprogramming. Antioxidants.

[50] Wang J., Yin N., Deng Y., Wei Y., Huang Y., Pu X., Li L., Zheng Y., Guo J., Yu J. (2016). Ascorbic Acid Protects against Hypertension through Downregulation of ACE1 Gene Expression Mediated by Histone Deacetylation in Prenatal Inflammation Induced Offspring. Sci. Rep..

[51] Koeners M.P., Racasan S., Koomans H.A., Joles J.A., Braam B. (2007). Nitric oxide, superoxide and renal blood flow autoregulation in SHR after perinatal L-arginine and antioxidants. Acta Physiol..

[52] Silva L.B.A.R., Pinheiro-Castro N., Novaes G.M., Pascoal G.F.L., Ong T.P. (2019). Bioactive food compounds, epigenetics and chronic disease prevention: Focus on early-life interventions with polyphenols. Food Res. Int..

[53] Care A.S., Sung M.M., Panahi S., Gragasin F.S., Dyck J.R., Davidge S.T., Bourque S.L. (2016). Perinatal Resveratrol Supplementation to Spontaneously Hypertensive Rat Dams Mitigates the Development of Hypertension in Adult Offspring. Hypertension.

[54] Zou T., Chen D., Yang Q., Wang B., Zhu M.J., Nathanielsz P.W., Du M. (2017). Resveratrol supplementation of high-fat diet-fed pregnant mice promotes brown and beige adipocyte development and prevents obesity in male offspring. J. Physiol..

[55] Tain Y.L., Huang L.T., Lee C.T., Chan J.Y., Hsu C.N. (2015). Maternal citrulline supplementation prevents prenatal NG-nitro-L-arginine-methyl ester (L-NAME)-induced programmed hypertension in rats. Biol. Reprod..

[56] Tai I.H., Sheen J.M., Lin Y.J., Yu H.R., Tiao M.M., Chen C.C., Huang L.T., Tain Y.L. (2016). Maternal N-acetylcysteine therapy regulates hydrogen sulfide-generating pathway and prevents programmed hypertension in male offspring exposed to prenatal dexamethasone and postnatal high-fat diet. Nitric Oxide.

[57] Hardeland R., Tan D.X., Reiter R.J. (2009). Kynuramines, metabolites of melatonin and other indoles: The resurrection of an almost forgotten class of biogenic amines. J. Pineal Res..

[58] Back K., Tan D.X., Reiter R.J. (2016). Melatonin biosynthesis in plants: Multiple pathways catalyze tryptophan to melatonin in the cytoplasm or chloroplasts. J. Pineal Res..

[59] Klein D.C. (2007). Arylalkylamine N-acetyltransferase: “the Timezyme”. J. Biol. Chem..

[60] Waldhauser F., Ehrhart B., Förster E. (1993). Clinical aspects of the melatonin action: Impact of development, aging, and puberty, involvement of melatonin in psychiatric disease and importance of neuroimmunoendocrine interactions. Experientia.

[61] Tan D.X., Manchester L.C., Esteban-Zubero E., Zhou Z., Reiter R.J. (2015). Melatonin as a Potent and Inducible Endogenous Antioxidant: Synthesis and Metabolism. Molecules.

[62] Acuña-Castroviejo D., Escames G., Venegas C., Díaz-Casado M.E., Lima-Cabello E., López L.C., Rosales-Corral S., Tan D.X., Reiter R.J. (2014). Extrapineal melatonin: Sources, regulation, and potential functions. Cell. Mol. Life Sci..

[63] Reiter R.J., Tan D.X., Korkmaz A., Rosales-Corral S.A. (2014). Melatonin and stable circadian rhythms optimize maternal, placental and fetal physiology. Hum. Reprod. Update.

[64] Ekmekcioglu C. (2006). Melatonin receptors in humans: Biological role and clinical relevance. Biomed. Pharmacother..

[65] Nosjean O., Ferro M., Coge F., Beauverger P., Henlin J.M., Lefoulon F., Fauchere J.L., Delagrange P., Canet E., Boutin J.A. (2000). Identification of the melatonin-binding site MT3 as the quinone reductase 2. J. Biol. Chem..

[66] Kennaway D.J., Stamp G.E., Goble F.C. (1992). Development of melatonin production in infants and the impact of prematurity. J. Clin. Endocrinol. Metab..

[67] Tain Y.L., Huang L.T., Hsu C.N., Lee C.T. (2014). Melatonin therapy prevents programmed hypertension and nitric oxide deficiency in offspring exposed to maternal caloric restriction. Oxidative Med. Cell Longev..

[68] Tain Y.L., Chen C.C., Sheen J.M., Yu H.R., Tiao M.M., Kuo H.C., Huang L.T. (2014). Melatonin attenuates prenatal dexamethasone-induced blood pressure increase in a rat model. J. Am. Soc. Hypertens..

[69] Tain Y.L., Lee C.T., Chan J.Y., Hsu C.N. (2016). Maternal melatonin or N-acetylcysteine therapy regulates hydrogen sulfide-generating pathway and renal transcriptome to prevent prenatal N^G^-Nitro-L-arginine-methyl ester (L-NAME)-induced fetal programming of hypertension in adult male offspring. Am. J. Obstet. Gynecol..

[70] Tain Y.L., Lin Y.J., Chan J.Y.H., Lee C.T., Hsu C.N. (2017). Maternal melatonin or agomelatine therapy prevents programmed hypertension in male offspring of mother exposed to continuous light. Biol. Reprod..

[71] Hansell J.A., Richter H.G., Camm E.J., Herrera E.A., Blanco C.E., Villamor E., Patey O.V., Lock M.C., Trafford A.W., Galli G.L.J. (2022). Maternal melatonin: Effective intervention against developmental programming of cardiovascular dysfunction in adult offspring of complicated pregnancy. J. Pineal Res..

[72] Gao L., Zhao Y.C., Liang Y., Lin X.H., Tan Y.J., Wu D.D., Li X.Z., Ye B.Z., Kong F.Q., Sheng J.Z. (2016). The impaired myocardial ischemic tolerance in adult offspring of diabetic pregnancy is restored by maternal melatonin treatment. J. Pineal Res..

[73] Lapa Neto C.J.C., de Melo I.M.F., Alpiovezza P.K.B.M., de Albuquerque Y.M.L., Francisco Soares A., Teixeira Á.A.C., Wanderley-Teixeira V. (2023). Melatonin associated with a high-fat diet during pregnancy and lactation prevents liver changes in the offspring. Gen. Comp. Endocrinol..

[74] Tain Y.L., Leu S., Wu K.L., Lee W.C., Chan J.Y. (2014). Melatonin prevents maternal fructose intake-induced programmed hypertension in the offspring: Roles of nitric oxide and arachidonic acid metabolites. J. Pineal Res..

[75] Ferreira D.S., Amaral F.G., Mesquita C.C., Barbosa A.P., Lellis-Santos C., Turati A.O., Santos L.R., Sollon C.S., Gomes P.R., Faria J.A. (2012). Maternal melatonin programs the daily pattern of energy metabolism in adult offspring. PLoS ONE.

[76] Kett M.M., Denton K.M. (2011). Renal programming: Cause for concern?. Am. J. Physiol. Regul. Integr. Comp. Physiol..

[77] Bertram J.F., Douglas-Denton R.N., Diouf B., Hughson M., Hoy W. (2011). Human nephron number: Implications for health and disease. Pediatr. Nephrol..

[78] Nenov V.D., Taal M., Sakharova O.V., Brenner B.M. (2000). Multi-hit nature of chronic renal disease. Curr. Opin. Nephrol. Hypertens..

[79] Tain Y.L., Huang L.T., Chan J.Y. (2014). Transcriptional regulation of programmed hypertension by melatonin: An epigenetic perspective. Int. J. Mol. Sci..

[80] Rochette L., Lorin J., Zeller M., Guilland J.C., Lorgis L., Cottin Y., Vergely C. (2013). Nitric oxide synthase inhibition and oxidative stress in cardiovascular diseases: Possible therapeutic targets?. Pharmacol. Ther..

[81] Tain Y.L., Kao Y.H., Hsieh C.S., Chen C.C., Sheen J.M., Lin I.C., Huang L.T. (2010). Melatonin blocks oxidative stress-induced increased asymmetric dimethylarginine. Free Radic. Biol. Med..

[82] Zhu Z., Cao F., Li X. (2019). Epigenetic Programming and Fetal Metabolic Programming. Front. Endocrinol..

[83] Godfrey K.M., Costello P.M., Lillycrop K.A. (2016). Development, Epigenetics and Metabolic Programming. Nestle. Nutr. Inst. Workshop Ser..

[84] Peschke E., Bähr I., Mühlbauer E. (2013). Melatonin and pancreatic islets: Interrelationships between melatonin, insulin and glucagon. Int. J. Mol. Sci..

[85] Galano A., Tan D.X., Reiter R.J. (2013). On the free radical scavenging activities of melatonin’s metabolites, AFMK and AMK. J. Pineal Res..

[86] Reiter R.J., Tan D.X., Manchester L.C., Qi W. (2001). Biochemical reactivity of melatonin with reactive oxygen and nitrogen species: A review of the evidence. Cell Biochem. Biophys..

[87] Reiter R.J., Rosales-Corral S., Tan D.X., Jou M.J., Galano A., Xu B. (2017). Melatonin as a mitochondria-targeted antioxidant: One of evolution’s best ideas. Cell. Mol. Life Sci..

[88] Wu T.H., Kuo H.C., Lin I.C., Chien S.J., Huang L.T., Tain Y.L. (2014). Melatonin prevents neonatal dexamethasone induced programmed hypertension: Histone deacetylase inhibition. J. Steroid Biochem. Mol. Biol..

[89] Tsai C.C., Lin Y.J., Yu H.R., Sheen J.M., Lin I.C., Lai Y.J., Tain Y.L., Huang L.T., Tiao M.M. (2018). Regulation of Leptin Methylation Not via Apoptosis by Melatonin in the Rescue of Chronic Programming Liver Steatosis. Int. J. Mol. Sci..

[90] Lui C.C., Hsu M.H., Kuo H.C., Chen C.C., Sheen J.M., Yu H.R., Tiao M.M., Tain Y.L., Chang K.A., Huang L.T. (2015). Effects of melatonin on prenatal dexamethasone-induced epigenetic alterations in hippocampal morphology and reelin and glutamic acid decarboxylase 67 levels. Dev. Neurosci..

[91] Cantó C., Auwerx J. (2009). PGC-1α, SIRT1 and AMPK, an energy sensing network that controls energy expenditure. Curr. Opin. Lipidol..

[92] Hardeland R. (2017). Melatonin and the electron transport chain. Cell. Mol. Life Sci..

[93] Gupta S.K., Vyavahare S., Duchesne Blanes I.L., Berger F., Isales C., Fulzele S. (2023). Microbiota-derived tryptophan metabolism: Impacts on health, aging, and disease. Exp. Gerontol..

[94] Bonmatí-Carrión M.Á., Rol M.A. (2023). Melatonin as a Mediator of the Gut Microbiota-Host Interaction: Implications for Health and Disease. Antioxidants.

[95] Khan M.T., Zohair M., Khan A., Kashif A., Mumtaz S., Muskan F. (2025). From Gut to Brain: The roles of intestinal microbiota, immune system, and hormones in intestinal physiology and gut-brain-axis. Mol. Cell. Endocrinol..

[96] Tain Y.L., Hsu C.N. (2024). Nutritional Approaches Targeting Gut Microbiota in Oxidative-Stress-Associated Metabolic Syndrome: Focus on Early Life Programming. Nutrients.

[97] Hsu C.N., Yang H.W., Hou C.Y., Chang-Chien G.P., Lin S., Tain Y.L. (2021). Melatonin Prevents Chronic Kidney Disease-Induced Hypertension in Young Rat Treated with Adenine: Implications of Gut Microbiota-Derived Metabolites. Antioxidants.

[98] Leelaviwat N., Mekraksakit P., Cross K.M., Landis D.M., McLain M., Sehgal L., Payne J.D. (2022). Melatonin: Translation of Ongoing Studies Into Possible Therapeutic Applications Outside Sleep Disorders. Clin. Ther..

[99] Sugden D. (1983). Psychopharmacological effects of melatonin in mouse and rat. J. Pharmacol. Exp. Ther..

[100] Reagan-Shaw S., Nihal M., Ahmad N. (2008). Dose translation from animal to human studies revisited. FASEB J..

[101] Andersen L.P., Gögenur I., Rosenberg J., Reiter R.J. (2016). Pharmacokinetics of Melatonin: The Missing Link in Clinical Efficacy?. Clin. Pharmacokinet..

[102] Ives C.W., Sinkey R., Rajapreyar I., Tita A.T.N., Oparil S. (2020). Preeclampsia-Pathophysiology and Clinical Presentations: JACC State-of-the-Art Review. J. Am. Coll. Cardiol..

[103] Lanoix D., Guérin P., Vaillancourt C. (2012). Placental melatonin production and melatonin receptor expression are altered in preeclampsia: New insights into the role of this hormone in pregnancy. J. Pineal Res..

[104] Zeng K., Gao Y., Wan J., Tong M., Lee A.C., Zhao M., Chen Q. (2016). The reduction in circulating levels of melatonin may be associated with the development of preeclampsia. J. Hum. Hypertens..

[105] Hobson S.R., Gurusinghe S., Lim R., Alers N.O., Miller S.L., Kingdom J.C., Wallace E.M. (2018). Melatonin improves endothelial function in vitro and prolongs pregnancy in women with early-onset preeclampsia. J. Pineal Res..

[106] Lanoix D., Lacasse A.A., Reiter R.J., Vaillancourt C. (2013). Melatonin: The watchdog of villous trophoblast homeostasis against hypoxia/reoxygenation-induced oxidative stress and apoptosis. Mol. Cell. Endocrinol..

[107] Burton G.J., Jauniaux E. (2018). Pathophysiology of placental-derived fetal growth restriction. Am. J. Obstet. Gynecol..

[108] Blok E.L., Burger R.J., Bergeijk J.E.V., Bourgonje A.R., Goor H.V., Ganzevoort W., Gordijn S.J. (2024). Oxidative stress biomarkers for fetal growth restriction in umbilical cord blood: A scoping review. Placenta.

[109] Joo E.H., Kim Y.R., Kim N., Jung J.E., Han S.H., Cho H.Y. (2021). Effect of Endogenic and Exogenic Oxidative Stress Triggers on Adverse Pregnancy Outcomes: Preeclampsia, Fetal Growth Restriction, Gestational Diabetes Mellitus and Preterm Birth. Int. J. Mol. Sci..

[110] Berbets A.M., Davydenko I.S., Barbe A.M., Konkov D.H., Albota O.M., Yuzko O.M. (2021). Melatonin 1A and 1B Receptors’ Expression Decreases in the Placenta of Women with Fetal Growth Restriction. Reprod. Sci..

[111] Chen Y.C., Sheen J.M., Tiao M.M., Tain Y.L., Huang L.T. (2013). Roles of melatonin in fetal programming in compromised pregnancies. Int. J. Mol. Sci..

[112] Nakamura Y., Tamura H., Kashida S., Takayama H., Yamagata Y., Karube A., Sugino N., Kato H. (2001). Changes of serum melatonin level and its relationship to feto-placental unit during pregnancy. J. Pineal Res..

[113] Li L., Zhou L., Li W., Shi F., Feng X., Zhuang J. (2025). Oxidative stress biomarkers in fetal growth restriction: A systematic review and meta-analysis. Arch. Gynecol. Obstet..

[114] (2025). ClinicalTrials.gov. https://clinicaltrials.gov/.

[115] Torres-Torres J., Monroy-Muñoz I.E., Perez-Duran J., Solis-Paredes J.M., Camacho-Martinez Z.A., Baca D., Espino-Y-Sosa S., Martinez-Portilla R., Rojas-Zepeda L., Borboa-Olivares H. (2024). Cellular and Molecular Pathophysiology of Gestational Diabetes. Int. J. Mol. Sci..

[116] Zheng C., Dalla Man C., Cobelli C., Groop L., Zhao H., Bale A.E., Shaw M., Duran E., Pierpont B., Caprio S. (2015). A common variant in the MTNR1b gene is associated with increased risk of impaired fasting glucose (IFG) in youth with obesity. Obesity.

[117] Zhan Y., Li C., Gao Q., Chen J., Yu S., Liu S.G. (2015). Association between the rs4753426 polymorphism in MTNR1B with fasting plasma glucose level and pancreatic b-cell function in gestational diabetes mellitus. Genet. Mol. Res..

[118] Li C., Zhou Y., Qiao B., Xu L., Li Y., Li C. (2019). Association Between a Melatonin Receptor IB Genetic Polymorphism and Its Protein Expression in Gestational Diabetes Mellitus. Reprod. Sci..

[119] Siddhi J., Sherkhane B., Kalavala A.K., Arruri V., Velayutham R., Kumar A. (2022). Melatonin prevents diabetes-induced nephropathy by modulating the AMPK/SIRT1 axis: Focus on autophagy and mitochondrial dysfunction. Cell Biol. Int..

[120] Chung S., Yeh T., Wu C.H. (2017). Trend and pattern of herb and supplement use among pregnant women in the United States: Findings from the 2002, 2007, and 2012 US National Health Interview Surveys. Am. J. Obstet. Gynecol..

[121] Chavatte-Palmer P., Tarrade A., Rousseau-Ralliard D. (2016). Diet before and during Pregnancy and Offspring Health: The Importance of Animal Models and What Can Be Learned from Them. Int. J. Environ. Res. Public Health.

[122] Furukawa S., Kuroda Y., Sugiyama A. (2014). A comparison of the histological structure of the placenta in experimental animals. J. Toxicol. Pathol..

[123] Morel O., Laporte-Broux B., Tarrade A., Chavatte-Palmer P. (2012). The use of ruminant models in biomedical perinatal research. Theriogenology.

[124] Sengupta P. (2013). The Laboratory Rat: Relating Its Age with Human’s. Int. J. Prev. Med..

[125] Krege J.H., Hodgin J.B., Hagaman J.R., Smithies O. (1995). A noninvasive computerized tail-cuff system for measuring blood pressure in mice. Hypertension.

[126] Freeman M.P., Sosinsky A.Z., Moustafa D., Viguera A.C., Cohen L.S. (2016). Supplement use by women during pregnancy: Data from the Massachusetts General Hospital National Pregnancy Registry for Atypical Antipsychotics. Arch. Womens Ment. Health.

[127] Ziaei S., Hasani M., Malekahmadi M., Daneshzad E., Kadkhodazadeh K., Heshmati J. (2024). Effect of melatonin supplementation on cardiometabolic risk factors, oxidative stress and hormonal profile in PCOS patients: A systematic review and meta-analysis of randomized clinical trials. J. Ovarian Res..

[128] Albzea W., Almonayea L., Aljassar M., Atmeh M., Al Sadder K., AlQattan Y., Alhajaji R., AlNadwi H., Alnami I., Alhajaji F. (2023). Efficacy and Safety of Preoperative Melatonin for Women Undergoing Cesarean Section: A Systematic Review and Meta-Analysis of Randomized Placebo-Controlled Trials. Medicina.

[129] Wu Y., Huang W., Tang L., Feng Y., Chen H., Pan M., Peng J., Li C., Wang H. (2025). Melatonin improved the outcomes of women with ART: A systematic review and meta-analysis of randomized trials. Front. Reprod. Health.

[130] Tang H., Hao J., Xu B., Wang Y., Li Y., Zhao J. (2025). Melatonin supplementation and outcomes of assisted reproductive technology: A systematic review and meta-analysis. BMC Pregnancy Childbirth.

[131] Ossenia R.A., Rata P., Bogdan A., Warneta J.M., Touitou Y. (2000). Evidence of prooxidant and antioxidant action of melatonin on human liver cell line HepG2. Life Sci..

[132] Tain Y.L., Hsu C.N. (2024). Maternal Polyphenols and Offspring Cardiovascular-Kidney-Metabolic Health. Nutrients.

